# HTLV-1 proviral load levels in adult T-cell leukemia/lymphoma (ATL) in Bahia, Brazil

**DOI:** 10.1186/1742-4690-8-S1-A91

**Published:** 2011-06-06

**Authors:** Isabela Archanjo, Marcelo Magalhães, Everton S Batista, Kiyoshi Fukutani, Achilea Bittencourt, Lourdes Farre

**Affiliations:** 1 Laboratory of Experimental Pathology, CPQGM, FIOCRUZ, Salvador, Bahia, 40296710, Brazil; 2 Laboratory of Immunoparasitology, CPQGM, FIOCRUZ, Salvador, Bahia, 40296710, Brazil; 3 Department of Pathology, HUPES, Federal University of Bahia, Salvador, Bahia, 40296710, Brazil

## 

In human T-cell leukemia virus type 1 (HTLV-1) infection, high levels of proviral load (PVL) have been associated with the development of HTLV-1-associated myelopathy/tropical spastic paraparesis(HAM/TSP) and infective dermatitis associated with HTLV-1 (IDH). Few data are available on PVL in the different clinical types of adult T-cell leukemia/lymphoma (ATL), a malignancy associated with HTLV-1. PVL assessment may constitute a useful tool for classifying types of ATL. The objective of this study was to assess the PVL levels of patients with different clinical types of ATL. The study included 39 samples from patients with the following clinical subtypes of ATL: acute (n = 11), chronic (n = 11), smoldering (n = 10), lymphoma (n = 5) and primary cutaneous tumoral (n = 2). PVL was quantified using quantitative real-time PCR, taking the tax region of provirus into consideration and using β-globulin as the reference gene. PVL levels were highest in the chronic and acute subtypes, but there was no statistically significant difference between these two subtypes (p = 0.6936). In smoldering ATL, PVL levels were lower compared to the levels found in the acute (p = 0.0124) and chronic (p = 0.0067) subtypes. In the primary cutaneous tumoral and lymphoma subtypes, PVL levels were intermediate but there were no statistically significant differences between these and the other clinical subtypes. The present results suggest that PVL levels in ATL are related to the lymphocytosis observed in the acute and chronic subtypes, but appear to be unrelated to the aggressiveness of the disease.

